# Microbiological findings of the maternal periodontitis associated to low birthweight

**DOI:** 10.31744/einstein_journal/2020AO4209

**Published:** 2020-08-28

**Authors:** Mariana Cedraz de Oliveira, Isaac Suzart Gomes-Filho, Andreas Stöcker, Laerte Oliveira Barreto, André do Nascimento Santos, Simone Seixas da Cruz, Johelle Santana Passos-Soares, Michelle Miranda Lopes Falcão, José Roberto Cardoso Meireles, Gregory John Seymour, Roberto Meyer, Soraya Castro Trindade

**Affiliations:** 1 Universidade Federal da Bahia SalvadorBA Brazil Universidade Federal da Bahia , Salvador , BA , Brazil .; 2 Universidade Estadual de Feira de Santana Feira de SantanaBA Brazil Universidade Estadual de Feira de Santana , Feira de Santana , BA , Brazil .; 3 Universidade Federal do Recôncavo da Bahia Santo Antônio de JesusBA Brazil Universidade Federal do Recôncavo da Bahia , Santo Antônio de Jesus , BA , Brazil .; 4 University of Otago DunedinNZ New Zeland University of Otago , Dunedin , NZ , New Zeland .

**Keywords:** Periodontitis, Infant, low birth weight, Biofilms, Microbiota, Pregnancy

## Abstract

**Objective:**

To determine the association between the presence of periodontal pathogens and low birthweight.

**Methods:**

This observational and case-control study consisted of mothers of infants weighing <2,500g (Group A), and mothers of newborns weighing ≥2,500g (Group B), born at *Hospital da Mulher* in Feira de Santana (BA), Brazil. A semi-structured questionnaire covering demographic data, gestational history and aspects related to general and oral health was employed postpartum. Following a complete periodontal examination, biofilm samples were collected at six sites in the mouth. The participants were further categorized in terms of presence or absence of periodontitis. Differences between the groups were determined using Pearson’s χ ^2^ test, odds ratio, and confidence intervals were obtained using the Mantel-Haenszel test.

**Results:**

*Aggregatibacter actinomycetemcomitans, Porphyromonas gingivalis, Treponema denticola, Tannerella forsythia* and *Prevotella intermedia* were detected by polymerase chain reaction. A total of 303 postpartum women were evaluated, 224 (73.9%) in Group B. Statistically significant differences between the groups were found for age, body mass index and history of previous low birthweight babies. Group A had a higher frequency of periodontitis (33.34%) than Group B (16.22%). *P. gingivalis* and *P. intermedia* were detected more frequently among women with periodontitis (74.19% and 88.70%, respectively).

**Conclusion:**

In this population, there was no association between the presence of maternal periodontal pathogens and the occurrence of low birthweight infants.

## INTRODUCTION

Although it is well established that increases in hormone levels during pregnancy may cause significant exacerbation of gingivitis, ^( 1 )^ it was not until the 1990s, that evidence regarding associations between maternal periodontitis and undesirable pregnancy outcomes, such as preterm birth (PTB) and low birthweight (LBW), began to emerge. ^( 2 , 3 )^ Modest but statistically significant associations ^( 4 )^ were found in observational studies, even after controlling for factors that contribute to both periodontitis and adverse pregnancy outcomes. ^( 5 )^ However, intervention studies failed to show that periodontal therapy reduced the overall rates of PTB and LBW. ^( 6 )^

This lack of consensus may be attributed to variations in the populations evaluated, presence of potential confounding factors, and discrepancies in the way periodontitis is defined in different studies. ^( 7 )^ Nonetheless, despite the different methods used, the prevalence of periodontal disease remains high among pregnant women. ^( 8 - 10 )^ Furthermore, the complexities involved in performing intervention studies (inappropriate timing and/or type of intervention, inclusion of patients whose risk is not modifiable, and transient bacteremia caused by periodontal treatment) warrants a more thorough understanding of the effects of treatment and improvement in periodontal health on the pathophysiology of PTB and LBW. ^( 4 )^

Several studies investigating the association between the presence of periodontopathogens and the risk of adverse pregnancy outcomes have been reported. ^( 5 , 11 - 13 )^ However, these reports are contradictory and divergent in terms of sample size, the number of biofilm collection sites, the technique used to detect pathogens, and the prevalence of periodontal disease in the populations studied. Therefore, the meticulous study of maternal periodontal pathogens as markers of risk for PTB and LBW remains crucially important.

Previous studies carried out in Northeastern region of Brazil have demonstrated an association between periodontitis and LBW. ^( 14 , 15 )^ We believe that studies assessing the relation between maternal periodontal pathogens and can broad current knowledge by providing a substantially larger sample size and allowing for the exploration of potential confounders.

## OBJECTIVE

To determine the association between the presence of maternal periodontal pathogens and low birthweight.

## METHODS

### Study design

The present case-control study consisted of a Case Group, with mothers of newborns whose birthweight was <2,500g (Group A), and a Control Group, mothers of children from the same hospital whose birthweight was ≥2,500g (Group B). In addition, these women were further grouped in accordance with their periodontal condition: presence of periodontitis (P), indicating clinically diagnosed periodontitis, or absence of periodontitis (NP), without periodontitis.

### Study sample

The participants were women who gave birth at the *Hospital Inácia Pinto dos Santos* , in the city of Feira de Santana, state of Bahia, Brazil, between June 2012 and June 2013.

The sample size calculation was based on the frequency of periodontitis among mothers in the Group A (57.8%) and those in the Group B (39%) in a previous study. ^( 14 )^ The minimum sample size (316) with 79 cases and 237 controls was determined using Epi Info™ software, assuming 95% of confidence level and 80% power, with a ratio of three controls for each case.

The study was approved by the *Universidade Estadual de Feira de Santana* Institutional Review Board (no. 152/2008), CAAE: 0151.0.059.000-08. All women signed the Informed Consent Form.

All participants were asked to complete a semi-structured questionnaire containing identification and demographic data, as well as information pertaining to obstetric history, lifestyle habits and oral health aspects.

Postpartum women with cardiovascular disease, type 2 *diabetes mellitus* , kidney disease, parathyroid disease, urolithiasis, bacterial vaginosis, congenital malformation of the fetus, and other factors that could affect pregnancy outcome ^( 5 )^ were not included. Mothers who required antibiotic prophylaxis for dental procedures or had undergone periodontal treatment during pregnancy were also excluded.

### Diagnosis of periodontitis and newborn birthweight

Each woman’s periodontal condition was evaluated by a single, trained dentist. The degree of agreement between the obtained result and the expected result in the evaluation of the clinical attachment level was measured by the Kappa coefficient (0.832 indicated almost perfect agreement) for examiner selection. The periodontal examination was performed in the hospital until 3 days after the delivery. The following clinical parameters were evaluated in six sites per tooth: probing depth, gingival recession, clinical attachment level and bleeding on probing. The examiner was blinded to the status of the participants at the time of clinical periodontal examination. Periodontitis was defined as three or more teeth with one or more sites presenting a probing depth ≥4mm, clinical attachment level ≥3mm, as well as bleeding on probing at the same site. ^( 16 )^

The hospital birth register was searched to obtain data on weight of each newborn. Each woman’s gestational outcome was evaluated in accordance with World Health Organization (WHO) criteria ^( 17 )^ with respect to LBW (<2,500g).

### Microbiological evaluation

The presence of five pathogens in the subgingival dental biofilm was evaluated: *Aggregatibacter actinomycetemcomitans* (Aa), *Porphyromonas gingivalis* (Pg), *Treponema denticola* (Td), *Tannerella forsythia* (Tf) and *Prevotella intermedia* (Pi). Co-infection, *i.e* ., the presence of three or more pathogens in a single participant, was also determined.

Following drying and isolation the subgingival dental biofilm was collected from the six sites of greatest probing depth in each individual using a Gracey curette (Hu-Friedy, Chicago, IL, USA).

Samples were pooled and stored in a single DNAse-free and RNAse-free microcentrifuge tube (Eppendorf, San Diego, CA, USA ^)^ with phosphate-buffered (PBS) saline sterile solution at -20°C until time of analysis.

Deoxyribonucleic acid (DNA) was extracted from each sample using a PureLink™ Genomic DNA Mini Kit (Invitrogen, Life Technologies, SP, BR) in accordance with manufacturer instructions. Polymerase chain reaction (PCR) was used to detect bacterial DNA using pairs of primers specific to the five periodontal pathogens: Aa, Pg, Td, Tf and Pi. ^( 18 , 19 )^ The presence of bacterial DNA was confirmed using a universal pair of primers that bind to the 16S rDNA sequence. ^( 18 )^

Each reaction was performed at a final volume of 25µL, containing 1x PCR buffer (Invitrogen, Life Technologies, SP,BR), 0.2mM of dNTP (Pharmacia Biotech, Piscataway, NJ, USA), 0.02U/µL of Platinum Taq polymerase (Invitrogen, Life Technologies, SP, BR), 0.3µM of each primer, 10ng of DNA Template and 14.15µL of RNAse-free H _2_ O. For denaturation, a thermocycler (Perkin Elmer, GeneAmp PCR System 2400, Norwalk, CT, USA) set at 94°C was used for 5 minutes. Next, reactions were annealed for 40 cycles at a suitable annealing temperature for each pair of primers. Finally, reactions were submitted to a temperature of 72°C for 4 minutes to complete DNA extension.

Amplification products were compared using a standard 100bp DNA ladder (Perkin Elmer, GeneAmp PCR System 2400, Norwalk, CT, USA). Electrophoresis was performed using 1.5% agarose gels in 1x TAE buffer (Perkin Elmer, GeneAmp PCR System 2400, Norwalk, CT, USA) stained with SYBR ^®^ Safe DNA Gel Stain (Perkin Elmer, GeneAmp PCR System 2400, Norwalk, CT, USA). Gels were photographed under a blue-light transilluminator (Kodak Digital Science System 120, Eastman Kodak Company, NY, USA).

### Statistical analysis

Simple frequencies and central trend measurements were calculated using the study sample’s sociodemographic, medical and lifestyle data. The groups were compared using the χ ^2^ distribution test with respect to categorical variables, with a significance level of 5% (p<0.05).

Odds ratio (OR) with a 95% confidence interval (CI) was used to evaluate the association between periodontitis and LBW, as well as any associations between the presence of any or a combination of the following periodontal pathogens Aa, Pg, Td, Tf, Pi, and LBW. The association between the co-infection condition and LBW was also evaluated. Stratified analysis and logistic regression analysis were conducted. The presence of effect-modifying covariables was investigated using the maximum likelihood ratio test (p<0.05). The presence of confounding covariables was evaluated utilizing backward strategy analysis. When any covariable varied more than 10% in the association measurement, it was confirmed as a confounder. Regardless of statistical confirmation, any covariable which is known to influence both periodontitis and LBW was also considered as a confounder. In sum, two types of models, crude and adjusted, were constructed to evaluate the associations studied.

The Hosmer-Lemeshow test was applied to verify goodness-of-fit. All data were analyzed using two software packages (Stata Data Analysis and Statistical Software, StataCorp LP, College Station, TX; SPSS – software, version 21.0, IBM, Armonk, NY ^)^ .

## RESULTS

The final sample consisted of 303 women who were evaluated and classified according to pregnancy outcome. Group A consisted of 79 (26.1%) mothers of newborns weighing <2,500g, while Group B had 224 (73.9%) mothers of newborns weighing ≥2,500g. The mean age of participants in Group A was 23±7.0 years (minimum 13 and maximum 57), and in the Group B, 24.6±6.1 years (minimum 13 and maximum 52) ( Table 1 ).

**Table 1:** Sociodemographic, life style and oral and general health condition of mothers of newborn with low birthweight

Characteristics	Group A	Group B	p value* ^†^
Age 13-17 and >36 years	30 (37.98)	35 (15.63)	0.000
Maternal schooling level (0-4 years of study)/ ^5^	13 (16.46)	38 (17.36)	0.856
Family income (0-1 minimum monthly salaries)/ ^3^	53 (67.95)	154 (69.36)	0.815
Household density (≥4 number of people)	25 (31.64)	63 (28.13)	0.553
Marital status/ ^3^			
Married/stable union	64 (82.05)	180 (81.08)	0.850
Single/widowed/divorced	14 (17.95)	42 (18.92)	
Smoking habit during pregnancy/ ^21^	5 (6.58)	7 (3.40)	0.240
Pre-pregnancy BMI≤18.5/ ^6^	11 (14.29)	7 (3.18)	0.000
Hypertension/ ^3^	17 (21.52)	38 (17.20)	0.394
Type 1 diabetes/ ^3^	3 (3.80)	11 (4.98)	0.670
Periodontitis/ ^3^	26 (33.34)	36 (16.22)	0.001
No tooth brusing after meals/ ^1^	1 (1.27)	10 (4.49)	0.189
No dental floss usage/ ^3^	50 (64.11)	146 (65.76)	0.791
No dentist visit during the pregnancy/ ^6^	57 (73.08)	150 (68.49)	0.449
No prenatal educational activity during pregnancy/ ^29^	63 (82.90)	145 (73.23)	0.094

Results expressed as n (%).

/ ^x^ missing data.

* p value: significance level ≤0.05; † χ ^2^ test. BMI: body mass index.

Statistical comparisons made among the cases and controls found homogeneity for the majority of the sociodemographic, medical and lifestyle characteristics evaluated, except for age (p=0.000) and pre-gestational body mass index (BMI) (p=0.000).

With respect to oral health, women in Group A had a statistically significantly higher frequency of periodontitis (33.34%) compared with those in Group B (16.22%) (p=0.001).

No statistically significant differences were observed between the groups in terms of the presence of periodontal pathogens. The frequencies of the periodontal pathogens in case and Group B were, respectively, as follows: 64.55% and 61.60% for Aa; 63.29% and 61.16% for Pg; 88.60% and 86.16% for Td; 79.74% and 75% for Pi; and 98.73% and 100% for Tf. When co-infection, *i.e* ., the concomitant presence of three or more periodontal pathogen species, was evaluated, the frequencies found in the Group A and Group B were 86.08% and 81.70%, respectively, which were not statistically significantly different.

Table 2 shows the distribution of the periodontal pathogens and the presence of periodontitis. All participants were regrouped to reflect the presence or absence of periodontitis (P and NP Groups). As expected, a statistically significant difference was detected with respect to some of the periodontal pathogens, as well as for coinfection, when comparing these two groups. *P. gingivalis* (p=0.023), and *P. intermedia* (p=0.009) were detected more frequently in women with periodontitis in comparison to those without the disease. The presence of three or more pathogens was also a more common finding in postpartum women with periodontitis in comparison to unaffected individuals (91.93% *versus* 80.26%; p=0.030).

**Table 2:** Distribution of five periodontopathogens in the subgingival biofilm samples, between the no periodontitis and periodontitis puerperal groups

Periodontopathogens	Group NP	Group P	p value* ^†^
*Agregatibacter actinomycetemcomitans* / ^3^	145 (60.92)	43 (69.35)	0.222
*Treponema denticola* / ^3^	139 (58.40)	46 (74.19)	0.023
*Prevotella intermedia* / ^3^	203 (85.29)	57 (91.93)	0.171
*Porphyromonas gingivalis* / ^3^	238 (100)	61 (98.38)	0.056
*Tannerella forsythia* / ^3^	173 (72.68)	55 (88.70)	0.009
Coinfection more than 2 species detected/ ^3^	191 (80.26)	57 (91.93)	0.030

Results expressed as n (%).

/ ^x^ missing data.

* p value: significance level ≤0.05; ^†^ χ ^2^ test.

**Figure 1:**
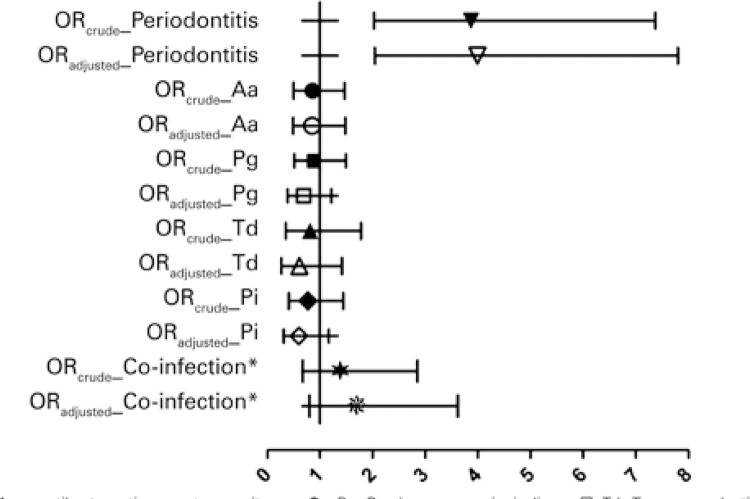
Association measurements, odds ratios, crude, and adjusted for age, pre-gestational body mass index and family income and respective 95% confidence intervals for the association between periodontopathogens, periodontitis and low birthweight * The concomitant presence of three or more periodontal pathogen species. OR: odds ratio; Aa: *Aggregatibacter actinomycetemcomitans;* Pg: *Porphyromonas gingivalis* ; Td: *Treponema denticola* ; Tf: *Tannerella forsythia;* Pi: *Prevotella intermedia* .

Figure 1 depicts the crude and adjusted OR association measurements with respect to the association between periodontitis and LBW, as well as between periodontal pathogens and LBW.

Crude model analysis revealed a statistically significant association only between periodontitis and LBW: OR _crude_ of 3.87, CI95%: 2.03-7.37. After adjusting for the covariables age, pre-gestational BMI and family income, a slight increase in the association measurement was observed (OR _adjusted_ of 3.99; CI95%: 2.04-7.80), with statistical significance maintained, revealing that mothers with periodontitis were four times more likely to give birth to children with LBW as compared to mothers without periodontitis.

With respect to comparisons between the presence of the periodontal pathogens Aa (OR _adjusted_ of 0.85; CI95%: 0.50-1.47), Pg (OR _adjusted_ of 0.69; CI95%: 0.39-1.22), Td (OR _adjusted_ of 0.61; CI95%: 0.27-1.42), Pi (OR _adjusted__=0._ 60; CI95%: 0.31-1.17) and LBW, as well as co-infection (OR _adjusted_ of 1.70; CI95%: 0.80-3.62)and LBW, no statistically significant associations were detected, even after adjusting for the same confounder covariables used in all adjusted analyses. Because the periodontal pathogen Tf was pervasive in the subgingival biofilm in both groups, it was impossible to obtain association measurements.

## DISCUSSION

The principal finding of the present study was an association between maternal periodontitis and LBW. However, no associations were found with respect to the presence of periodontal bacteria in the subgingival biofilm and LBW. Although these findings are in agreement with those reported by other authors, ^( 5 , 10 , 11 )^ some studies did detect an association between both clinical periodontitis, as well as the presence of periodontopathogens, and adverse gestational outcomes. ^( 2 , 20 , 21 )^

Despite this evidence surrounding the association between maternal periodontitis, as an exposure factor, and adverse gestational outcomes, the magnitude of the observed association has been modest. While the majority of observational studies have confirmed this association, interventional studies found no benefits with respect to gestational outcomes in terms of periodontal therapy. ^( 4 )^ In light of this controversy, the study of oral pathogens is crucial to the understanding of the nature of the challenge presented by periodontal disease.

The discrepancies between the present study and previous studies may be partially explained by variations in sample size, since most of these investigations had low numbers of participants. A total of 303 post-partum women were examined in the present study, which is more than most previous reports. ^( 11 - 13 , 22 )^ The relatively elevated power of the present study in comparison to other reports in the literature, contributes to the body of microbiological investigations focused on unfavorable gestational outcomes.

Another factor that may explain the apparent discrepancies is the study population considered in the present study, which was recruited from the Northeastern region in Brazil, where periodontal conditions are extremely poor and the prevalence of periodontal disease is high. ^( 23 )^ Nevertheless, another study performed in Southeastern Brazil ^( 5 )^ reported similar results, *i.e* ., an association between periodontitis and prematurity/LBW. These authors also found that none of the 39 species of bacteria evaluated in the subgingival biofilm of postpartum woman were associated with either of these gestational outcomes.

In addition, many of these studies evaluated a limited number of biofilm samples, usually two to four per participant, which may underestimate actual rates of periodontal infection. ^( 2 , 22 , 23 )^ By contrast, the present study collected six subgingival biofilm samples from each participant, which were obtained from the most compromised sites in each sextant.

Furthermore, it is interesting to note that these disparities may also be due to the clinical criteria utilized to define periodontitis, as well as study design, technique employed to evaluate the presence of periodontopathogens and the particular species of bacteria assessed.

A comparison of clinical and demographic characteristics of participants in the present study found homogeneity with respect to most aspects that represent risk factors for gestational complications. Nonetheless, some high-risk factors were observed at a higher frequency in mothers with LBW babies. Multivariate analysis was employed to adjust for age, pre-gestational BMI and family income in order to neutralize the effect of these confounding covariables in the group under study. However, no statistical significance was observed. This finding is in agreement with previous analyses, ^( 5 )^ which found no significant differences, even after adjusting for confounders, in the levels of periodontopathogens among women with no adverse gestational outcomes, as well as in those who delivered preterm and/or those who gave birth to newborns with LBW.

Despite the lack of statistical significance in this study for the association between maternal schooling level and LBW, it cannot be disregarded that education level and body perception influence the degree of knowledge about health care. ^( 24 )^

The present study found a high prevalence of periodontopathogens in both groups, which may be the result of what Dumitrescu et al., ^( 25 )^ called the “carrier state”, in which low quantities of periodontopathogens may be present in individuals with no clinical diagnosis of periodontitis. This further complicates the assessment of their role in this disease.

Since several species of periodontal bacteria are commonly found together in periodontal pockets, isolating the specific type responsible for the pathogenesis of this disease can be a complex task. ^( 25 )^ Due to the interaction and cooperation evidenced among diverse periodontopathogens, ^( 26 , 27 )^ the occurrence of coinfection among the women studied was also evaluated.

Our results demonstrate that the presence of three or more pathogens in pregnant mothers is not associated with LBW babies. However, unlike the OR analyses considering each pathogen separately, the association measurement with respect to co-infection approaches the level of significance.

The recent use of metagenomic sequencing techniques allows for the screening of genetic composition and the functional potential of a microbial community by providing a comprehensive overview of these communities in association with periodontal health and chronic periodontitis. ^( 28 )^ Furthermore, there may be several strains of each periodontopathogenic species with virulence factors of varying potential. The ability of each of these species to translocate, invade and colonize fetal tissues, as well as to evade host immune responses, determines its specific potential to contribute to adverse pregnancy outcomes. ^( 29 )^ Further studies are therefore necessary to investigate the heterogeneity of periodontal microbial communities in individuals.

It is well-known that bacteria alone are not capable of provoking severe tissue destruction in all individuals, which suggests that individuals have a variable capacity to adapt to a certain bacterial load within their biofilm. ^( 30 )^

The findings of the present study do not indicate that the presence of a specific periodontopathogen *per se* may be a plausible biological factor in the association between maternal periodontitis and the occurrence of babies with LBW. On the other hand, the present study not only reaffirms the association between maternal periodontitis and LBW baby, but it also broadens current knowledge by providing a substantially larger sample size, allowing for the exploration of potential confounders, increasing the number of biofilm collection sites and by considering a co-infection model in the analysis. Nonetheless, further studies are necessary to include a greater number of coinfection subgroups, employing larger samples.

## CONCLUSION

In this population, there was no association between the presence of periodontal pathogens and the occurrence of low birthweight infants.
