# *Erratum*: Microbiological findings of the maternal periodontitis associated to low birthweight

**DOI:** 10.31744/einstein_journal/2020AO4209E

**Published:** 2020-10-02

0000-0003-0393-2420 Mariana Cedraz de Oliveira^1^,

0000-0002-4270-8491 Isaac Suzart Gomes-Filho^2^,

0000-0001-8986-3133 Andreas Stöcker^1^,

0000-0001-9090-9063 Laerte Oliveira Barreto Neto^2^,

0000-0002-2042-7410 André do Nascimento Santos^2^,

0000-0003-2828-0887 Simone Seixas da Cruz^3^,

0000-0002-4541-1730 Johelle Santana Passos-Soares^1^,

0000-0002-0929-2324 Michelle Miranda Lopes Falcão^2^,

0000-0002-2008-4753 José Roberto Cardoso Meireles^2^,

0000-0001-7595-5651 Gregory John Seymour^4^,

0000-0002-4727-4805 Roberto Meyer^1^,

0000-0001-7125-9114 Soraya Castro Trindade^2^

^1^Universidade Federal da Bahia, Salvador, BA, Brazil.

^2^Universidade Estadual de Feira de Santana, Feira de Santana, BA, Brazil.

^3^Universidade Federal do Recôncavo da Bahia, Santo Antônio de Jesus, BA, Brazil.

^4^University of Otago, Dunedin, NZ, New Zeland.

In the manuscript “Microbiological findings of the maternal periodontitis associated to low birthweight”, DOI number: 10.31744/einstein_journal/2020AO4209, published at einstein (São Paulo). 2020;18:eAO4209, page 5, table 2:

**Stated:**

*Treponema denticola*/^3^

*Prevotella intermedia*//^3^

*Porphyromonas gingivalis*/^3^

*Tannerella forsythia*/^3^

**It should be read:**

*Porphyromonas gingivalis*/^3^

*Treponema denticola*/^3^

*Tannerella forsythia*/^3^

*Prevotella intermedia*/^3^

