# Supplementary material for: Microbiological findings of the maternal periodontitis associated to low birthweight
Source: Einstein (Sao Paulo). 2020 Aug 28;18:eAO4209. doi: 10.31744/einstein_journal/2020AO4209 (PMC7453829; doi:10.31744/einstein_journal/2020AO4209)
Supplement: Translation

# Translation: Achados microbiológicos da periodontite materna associados ao baixo peso ao nascer

**Authors:** Mariana Cedraz de Oliveira, Isaac Suzart Gomes-Filho, Andreas Stöcker, Laerte Oliveira Barreto, André do Nascimento Santos, Simone Seixas da Cruz, Johelle Santana Passos-Soares, Michelle Miranda Lopes Falcão, José Roberto Cardoso Meireles, Gregory John Seymour, Roberto Meyer, Soraya Castro Trindade

**Affiliations:** 1 Brasil Universidade Federal da Bahia, Salvador, BA, Brasil .; 2 Brasil Universidade Estadual de Feira de Santana, Feira de Santana, BA, Brasil .; 3 Brasil Universidade Federal do Recôncavo da Bahia, Santo Antônio de Jesus, BA, Brasil .; 4 New Zeland University of Otago, Dunedin, NZ, New Zeland .

**Keywords:** Periodontite, Recém-nascido de baixo peso, Biofilmes, Microbiota, Gravidez

## Abstract

**Objetivo:**

Determinar a associação entre a presença de patógenos periodontais e o baixo peso ao nascer.

**Métodos:**

Trata-se de estudo observacional e caso controle. Participaram mães de bebês com peso <2.500g (Grupo A) e de recém-nascidos com peso ≥2.500g (Grupo B) nascidos no Hospital da Mulher, em Feira de Santana, (BA). Dados demográficos, história gestacional e aspectos relacionados à saúde geral e bucal foram obtidos por meio de questionário semiestruturado, aplicado no período pós-parto. Após exame periodontal completo, as amostras de biofilme foram coletadas em seis locais na boca. As participantes foram também categorizadas quanto à presença ou ausência de periodontite. As diferenças entre os grupos foram obtidas pelo teste do χ ^2^ de Pearson, *odds ratio* e os intervalos de confiança pelo teste de Mantel-Haenszel.

**Resultados:**

*Aggregatibacter actinomycetemcomitans, Porphyromonas gingivalis, Treponema denticola, Tannerella forsythia* e *Prevotella intermedia* foram detectados por reação em cadeia de polimerase. Avaliaram-se 303 mulheres pós-parto, sendo 224 (73,9%) do Grupo B. Diferenças estatisticamente significativas entre os grupos foram observadas quanto à idade, ao índice de massa corporal e a história de partos prévios com bebês de baixo peso ao nascer. O Grupo A apresentou maior frequência de periodontite (33,34%) do que o Grupo B (16,22%). *P. gingivalis* e *P. intermedia* foram detectados com maior frequência em mulheres com periodontite (74,19% e 88,70%, respectivamente).

**Conclusão:**

Nessa população, não houve associação entre a presença de patógenos periodontais maternos e a ocorrência de bebês com baixo peso ao nascer.

## INTRODUÇÃO

Embora esteja bem estabelecido que o aumento dos níveis hormonais durante a gravidez possa causar exacerbação significativa da gengivite, ^( 1 )^ foi somente a partir da década de 1990 que começaram a ser observadas associações entre a periodontite materna e os desfechos indesejáveis da gestação, como parto pré-termo (PTT) e baixo peso ao nascer (BPN). ^( 2 , 3 )^ Foram encontradas associações moderadas, porém significativas, ^( 4 )^ em estudos observacionais, mesmo após o controle de fatores que contribuem tanto para a periodontite quanto para os desfechos adversos da gravidez. ^( 5 )^ Contudo, em estudos de intervenção, não foi possível mostrar que o tratamento periodontal resultasse em redução nas taxas gerais de PTT e BPN. ^( 6 )^

Essa falta de consenso pode ser atribuída a variações nas populações avaliadas, presença de possíveis fatores de confusão, e discrepâncias na definição da periodontite nos diferentes estudos. ^( 7 )^ No entanto, a despeito dos diferentes métodos utilizados, a prevalência da doença periodontal continua elevada nas gestantes. ^( 8 - 10 )^ Além disso, as complexidades envolvidas na realização de estudos de intervenção (momento inadequado e/ou tipo de intervenção, inclusão de pacientes cujo risco não é modificável, e bacteremia transitória causada pelo tratamento periodontal) justificam a compreensão mais aprofundada dos efeitos do tratamento e da melhoria da saúde periodontal na fisiopatologia do PTT e do BPN. ^( 4 )^

Foram relatados vários estudos que investigaram a associação entre a presença de periodontopatógenos e o risco de desfechos adversos da gestação. ^( 5 , 11 - 13 )^ No entanto, esses relatos são contraditórios e divergentes, em termos de tamanho da amostra, número de locais de coleta de biofilme, técnica usada na detecção de patógenos e prevalência de doença periodontal nas populações estudadas. Assim, é de crucial importância um estudo minucioso dos patógenos periodontais maternos como marcadores de risco para PTT e BPN.

Estudos anteriores realizados no Nordeste do Brasil demonstraram associação entre periodontite e BPN. ^( 14 , 15 )^ Acredita-se que estudos sobre a relação entre patógenos periodontais maternos e BPN possam ampliar o conhecimento atual, se realizados com uma amostra de tamanho significativamente maior e permitirem a exploração de potenciais fatores de confusão.

## OBJETIVO

Determinar a associação entre a presença de patógenos periodontais maternos e o baixo peso ao nascer.

## MÉTODOS

### Desenho do estudo

O presente estudo caso-controle consistiu em um Grupo Caso, com mães de recém-nascidos com peso ao nascer <2.500g (Grupo A), e um Grupo Controle, com mães de crianças nascidas no mesmo hospital cujo peso ao nascer era ≥2.500g (Grupo B). Além disso, essas mulheres foram agrupadas de acordo com sua condição periodontal: presença de periodontite (P), indicando periodontite diagnosticada clinicamente, ou ausência de periodontite (NP), sem periodontite.

### Amostra do estudo

As participantes eram mulheres que deram à luz no Hospital Inácia Pinto dos Santos, no município de Feira de Santana, estado da Bahia, entre junho de 2012 e junho de 2013.

O cálculo do tamanho da amostra foi baseado na frequência de periodontite entre as mães do Grupo A (57,8%) e entre as do Grupo B (39%), registrada em estudo anterior. ^( 14 )^ O tamanho mínimo da amostra (316), com 79 casos e 237 controles, foi determinado pelo software Epi Info™, assumindo-se nível de confiança de 95% e poder de 80%, com relação de três controles para cada caso.

O estudo foi aprovado pelo Comitê de Ética da Universidade Estadual de Feira de Santana (n.º 152/2008), CAAE: 0151.0.059.000-08. Todas as mulheres assinaram o Termo de Consentimento Livre e Esclarecido.

Todas as participantes foram convidadas a preencher um questionário semiestruturado contendo dados de identificação e demográficos, além de informações referentes à história obstétrica, ao hábitos de vida e aos aspectos de saúde bucal.

Não foram incluídas puérperas com doença cardiovascular, *diabetes mellitus* tipo 2, doença renal, doença da paratireoide, urolitíase, vaginose bacteriana, malformação congênita do feto, e outros fatores que poderiam afetar o desfecho da gravidez. ^( 5 )^ Também foram excluídas as mães que necessitaram de profilaxia com antibióticos para a realização de procedimentos odontológicos ou tinham sido submetidas a tratamento periodontal durante a gravidez.

### Diagnóstico de periodontite e peso do recém-nascido ao nascer

A condição periodontal de cada mulher foi avaliada por um único dentista treinado. O grau de concordância entre o resultado obtido e o esperado na avaliação do nível de inserção clínico foi medido pelo coeficiente de Kappa (0,832, que indicou concordância quase perfeita) por escolha do examinador. O exame periodontal foi realizado no hospital até 3 dias após o parto. Os seguintes parâmetros clínicos foram avaliados em seis sítios por dente: profundidade de sondagem, recessão gengival, nível de inserção clínica e sangramento à sondagem. O examinador foi mantido cego em relação ao grupo a que pertenciam as participantes no momento do exame clínico periodontal. A periodontite foi definida como três ou mais dentes com um ou mais sítios apresentando profundidade de sondagem ≥4mm, nível de inserção clínico ≥3mm, e sangramento à sondagem no mesmo sítio. ^( 16 )^

O registro de nascimento hospitalar foi consultado para obtenção dos dados de peso de cada recém-nascido. O desfecho gestacional de cada mulher foi avaliado de acordo com os critérios da Organização Mundial de Saúde (OMS) ^( 17 )^ no tocante ao BPN (<2.500g).

### Avaliação microbiológica

Foi avaliada a presença de cinco patógenos no biofilme dental subgengival: *Aggregatibacter actinomycetemcomitans* (Aa), *Porphyromonas gingivalis* (Pg), *Treponema denticola* (Td), *Tannerella forsythia* (Tf) and *Prevotella intermedia* (Pi). Foi também avaliada a coinfecção, ou seja, a presença de três ou mais patógenos em uma única participante.

Depois da secagem e do isolamento, coletou-se o biofilme dental subgengival de seis sítios de maior profundidade de sondagem em cada paciente usando uma cureta Gracey (Hu-Friedy, Chicago, IL, EUA).

As amostras foram reunidas e armazenadas em um único tubo de microcentrífuga livre de DNAse e RNAse (Eppendorf, San Diego, CA, EUA), com solução estéril salina tamponada com fosfato (PBS) a -20°C até o momento da análise.

Extraiu-se ácido desoxirribonucleico (DNA) de cada amostra, usando mini-kit PureLink™ Genomic DNA (Invitrogen, Life Technologies, SP, BR), de acordo com as instruções do fabricante. A reação em cadeia da polimerase (PCR) foi usada para detectar o DNA bacteriano, usando pares de *primers* específicos para os cinco patógenos periodontais: Aa, Pg, Td, Tf e Pi. ^( 18 , 19 )^ A presença do DNA bacteriano foi confirmada usando um par universal de *primers* que se ligam à sequência 16S rDNA. ^( 18 )^

Cada reação foi realizada com volume final de 25µL, contendo tampão de PCR 1x (Invitrogen, Life Technologies, SP,BR), 0,2mM de dNTP (Pharmacia Biotech, Piscataway, NJ, EUA), 0,02U/µL de Taq polimerase Platinum (Invitrogen, Life Technologies, SP, BR), 0,3µM de cada *primer* , 10ng de DNA molde e 14,15µL de H _2_ O livre de RNAse. Para desnaturação, foi usado um termociclador (Perkin Elmer, GeneAmp PCR System 2400, Norwalk, CT, EUA) fixado a 94°C, por 5 minutos. Em seguida, as reações foram recozidas por 40 ciclos a uma temperatura de recozimento adequada para cada par de *primers* . Por fim, as reações foram submetidas a uma temperatura de 72°C por 4 minutos para completar a extensão do DNA.

Os produtos da amplificação foram comparados usando uma escada de DNA padrão de 100pb (Perkin Elmer, GeneAmp PCR System 2400, Norwalk, CT, EUA). A eletroforese foi realizada usando géis de agarose a 1,5% em tampão TAE 1x (Perkin Elmer, GeneAmp PCR System 2400, Norwalk, CT, USA), corados com SYBR ^®^ Safe DNA Gel Stain (Perkin Elmer, GeneAmp PCR System 2400, Norwalk, CT, EUA). Os géis foram fotografados sob um transiluminador de luz azul (Kodak Digital Science System 120, Eastman Kodak Company, NY, EUA).

### Análise estatística

As frequências simples e as medidas de tendência central foram calculadas usando os dados sociodemográficos, clínicos e de estilo de vida da amostra do estudo. Os grupos foram comparados pelo teste de distribuição do χ ^2^ em relação às variáveis categóricas, com nível de significância de 5% (p<0,05).

*Odds ratio* (OR), com intervalo de confiança de 95% (IC95%), foi utilizada para avaliar a associação entre periodontite e BPN, bem como quaisquer associações entre a presença de qualquer agente ou uma combinação dos seguintes patógenos periodontais: Aa, Pg, Td, Tf, Pi e BPN. Também foi avaliada a associação entre a condição de coinfecção e o BPN. Foram realizadas análises estratificadas e de regressão logística. A presença de covariáveis modificadoras de efeito foi investigada pelo teste da razão de máxima verossimilhança (p<0,05). A presença de covariáveis de confusão foi considerada utilizando análise de estratégia reversa. Quando qualquer covariável variou mais de 10% na medida da associação, foi confirmada como fator de confusão. Independentemente da confirmação estatística, qualquer covariável conhecida por influenciar tanto a periodontite quanto o BPN também foi considerada fator de confusão. Em suma, dois tipos de modelos, não ajustado e ajustado, foram construídos para avaliar as associações estudadas.

O teste de Hosmer-Lemeshow foi aplicado para verificar a qualidade do ajuste. Todos os dados foram analisados usando dois pacotes de software (Stata Data Analysis and Statistical Software, StataCorp LP, College Station, TX; e SPSS – software, versão 21.0, IBM, Armonk, NY).

## RESULTADOS

A amostra final consistiu de 303 mulheres que foram avaliadas e classificadas de acordo com o desfecho da gravidez. O Grupo A tinha 79 (26,1%) mães de recém-nascidos com peso <2.500g, e o Grupo B tinha 224 (73,9%) mães de recém-nascidos com peso ≥2.500g. A média de idade das participantes do Grupo A era 23±7,0 anos (mínimo de 13 e máximo de 57), e, no Grupo B 24,6±6,1 anos (mínimo de 13 e máximo de 52) ( Tabela 1 ).

**Tabela 1:** Características sociodemográficas, estilo de vida e condição de saúde bucal e geral de mães de recém-nascidos com baixo peso ao nascer

Características	Grupo A	Grupo B	valor de p* ^†^
Idade 13-17 e >36 anos	30 (37,98)	35 (15,63)	0,000
Nível educacional materno de 0-4 anos de estudo/ ^5^ 0-4	13 (16,46)	38 (17,36)	0,856
Renda familiar de 0-1 salários mínimos mensais/ ^3^	53 (67,95)	154 (69,36)	0,815
Densidade familiar (≥4 pessoas)	25 (31,64)	63 (28,13)	0,553
Estado civil/ ^3^			
Casada/união estável	64 (82,05)	180 (81,08)	0,850
Solteira/viúva/divorciada	14 (17,95)	42 (18,92)	
Fumante durante a gravidez/ ^21^	5 (6,58)	7 (3,40)	0,240
IMC pré-gestacional ≤18,5/ ^6^	11 (14,29)	7 (3,18)	0,000
Hipertensão/ ^3^	17 (21,52)	38 (17,20)	0,394
Diabetes tipo 1/ ^3^	3 (3,80)	11 (4,98)	0,670
Periodontite/ ^3^	26 (33,34)	36 (16,22)	0,001
Não escovar os dentes após refeições/ ^1^	1 (1,27)	10 (4,49)	0,189
Não usar fio dental/ ^3^	50 (64,11)	146 (65,76)	0,791
Não ter feito consulta odontológica durante a gravidez/ ^6^	57 (73,08)	150 (68,49)	0,449
Sem atividade de educação pré-natal durante a gravidez/ ^29^	63 (82,90)	145 (73,23)	0,094

Resultados expressos por n (%).

/ ^x^ dados ausentes.

* valor de p: nível de significância ≤0,05; ^†^ teste χ ^2^ . IMC: índice de massa corporal.

As comparações estatísticas realizadas entre os casos e controles encontraram homogeneidade para a maioria das características sociodemográficas, clínicas e de estilo de vida avaliadas, com exceção da idade (p=0,000) e do índice de massa corporal (IMC) pré-gestacional (p=0,000).

Em relação à saúde bucal, as mulheres do Grupo A apresentaram frequência estatisticamente maior de periodontite (33,34%) em comparação com as do Grupo B (16,22%) (p=0,001).

Não foram observadas diferenças estatisticamente significativas entre os grupos quanto à presença de agentes patogênicos periodontais. As frequências dos agentes patogênicos periodontais nos Grupo A e Grupo B foram, respectivamente, 64,55% e 61,60% para Aa; 63,29% e 61,16% para Pg; 88,60% e 86,16% para Td; 79,74% e 75% para Pi; e 98,73% e 100% para Tf. Ao se avaliar a coinfecção, ou seja, a presença concomitante de três ou mais espécies de agentes patogênicos periodontais, as frequências encontradas nos Grupos A e B foram 86,08% e 81,70%, respectivamente, não tendo diferença estatística significativa.

A tabela 2 mostra a distribuição dos agentes patogênicos periodontais e a presença de periodontite. Todas as participantes foram reagrupadas para refletir a presença ou ausência de periodontite (Grupos P e NP). Conforme esperado, foi detectada diferença estatisticamente significativa em relação a alguns agentes patogênicos periodontais, bem como na coinfecção, ao comparar esses dois grupos. Foram detectados com maior frequência em mulheres com periodontite Pg (p=0,023) e Pi (p=0,009) em comparação àquelas sem a doença. A presença de três ou mais agentes patogênicos também foi achado mais comum em puérperas com periodontite em comparação com indivíduos não afetados (91,93% *versus* 80,26%; p=0,030).

**Tabela 2:** Distribuição dos cinco periodontopatógenos nas amostras de biofilme subgengivais, entre os grupos de puérperas sem periodontite e com periodontite

Agentes patogênicos periodontais	Grupo NP	Grupo P	Valor de p* ^†^
*Agregatibacter actinomycetemcomitans* / ^3^	145 (60,92)	43 (69,35)	0,222
*Porphyromonas gingivalis* / ^3^	139 (58,40)	46 (74,19)	0,023
*Treponema denticola* / ^3^	203 (85,29)	57 (91,93)	0,171
*Tannerella forsythia* / ^3^	238 (100)	61 (98,38)	0,056
*Prevotella intermedia* / ^3^	173 (72,68)	55 (88,70)	0,009
Coinfecção - mais de 2 espécies detectadas/ ^3^	191 (80,26)	57 (91,93)	0,030

Resultados expressos por n (%).

/ ^x^ dados ausentes.

* valor de p: nível de significância ≤0,05; ^†^ teste χ ^2^ .

A figura 1 mostra as medidas de associação estatística da OR não ajustada e ajustada, referentes à associação entre periodontite e BPN, bem como entre agentes patogênicos periodontais e BPN.

**Figura 1:** Medidas de associação estatística, *odds ratio* não ajustadas e ajustadas para idade, índice de massa corporal pré-gestacional e renda família, e os respectivos intervalos de confiança de 95% para a associação entre periodontopatógenos, periodontite e baixo peso ao nascer * Presença concomitante de três ou mais espécies de agentes patogênicos periodontais. OR: *odds ratio* ; Aa: *Aggregatibacter actinomycetemcomitans;* Pg: *Porphyromonas gingivalis* ; Td: *Treponema denticola;* Tf: *Tannerella forsythia;* Pi: *Prevotella intermedia.*

A análise do modelo não ajustado revelou associação estatisticamente significativa apenas entre periodontite e BPN: OR _não ajustada_ de 3,87 e IC95%: 2,03-7,37. Após o ajuste para as covariáveis idade, IMC pré-gestacional e renda familiar, observou-se pequeno aumento na medida da associação estatística (OR _ajustada_ de 3,99 e IC95%: 2,04-7,80), com significância estatística mantida, revelando que as mães com periodontite tiveram quatro vezes mais chances de dar à luz crianças com BPN do que as mães sem periodontite.

No tocante às comparações entre a presença dos patógenos periodontais Aa (OR _ajustada__d_ e 0,85; IC95%: 0,50-1,47), Pg (OR _ajustada_ de 0,69; IC95%: 0,39-1,22), Td (OR _ajustada_ de 0,61; IC95%: 0,27-1,42), Pi (OR _ajustada_ de 0,60; IC95%: 0,31-1,17) e BPN, assim como coinfecção (OR _ajustada_ de 1,70; IC95% [0,80 a 3,62]) e BPN, nenhuma associação estatisticamente significativa foi detectada, mesmos após ajuste para as mesmas covariáveis de confusão usadas em todas as análises ajustadas. Como o agente patogênico periodontal Tf estava presente no biofilme subgengival de ambos os grupos, não foi possível obter medidas de associação estatística.

## DISCUSSÃO

O principal achado do presente estudo foi uma associação entre periodontite materna e BPN. No entanto, não foram encontradas associações entre a presença de bactérias periodontais no biofilme subgengival e BPN. Embora esses achados estejam de acordo com os relatados por outros autores, ^( 5 , 10 , 11 )^ alguns estudos detectaram associação entre periodontite clínica, presença de periodontopatógenos e desfechos gestacionais adversos. ^( 2 , 20 , 21 )^

Apesar dessas evidências referentes à associação entre periodontite materna, como fator de exposição, e desfechos gestacionais adversos, a magnitude da associação observada tem sido modesta. Embora a maioria dos estudos observacionais tenha confirmado essa associação, não foram encontrados benefícios referentes aos desfechos gestacionais nos estudos de intervenção, em termos de terapia periodontal. ^( 4 )^ À luz dessa controvérsia, o estudo dos agentes patogênicos orais é crucial para a compreensão da natureza do desafio apresentado pela doença periodontal.

As discrepâncias entre este estudo e os anteriores podem ser parcialmente explicadas por variações no tamanho da amostra, uma vez que a maioria dessas investigações teve baixo número de participantes. Um total de 303 puérperas foram examinadas no presente estudo, ou seja, um número maior do que a da maioria dos relatórios anteriores. ^( 11 - 13 , 22 )^ O poder estatístico relativamente elevado do presente estudo, em comparação com outros relatos da literatura, serve para contribuir para o corpo de investigações microbiológicas focadas em desfechos gestacionais desfavoráveis.

Outro fator que pode explicar as aparentes discrepâncias é o fato de a população estudada neste estudo ter sido recrutada no Nordeste do Brasil, onde as condições periodontais são extremamente precárias, e a prevalência de doença periodontal é alta. ^( 23 )^ No entanto, outro estudo realizado no Sudeste do Brasil ^( 5 )^ relatou resultados semelhantes, ou seja, uma associação entre periodontite e prematuridade/BPN. Esses autores também descobriram que nenhuma das 39 espécies de bactérias avaliadas no biofilme subgengival das puérperas estava associada a qualquer um desses desfechos gestacionais.

Além disso, muitos desses estudos avaliaram um número limitado de amostras de biofilme (geralmente duas a quatro por participante), o que pode subestimar as taxas reais de infecção periodontal. ^( 2 , 22 , 23 )^ Por outro lado, o presente estudo coletou seis amostras de biofilme subgengival de cada participante, que foram obtidas nos locais mais comprometidos em cada sextante.

É interessante notar que essas disparidades também podem ser decorrentes dos critérios clínicos utilizados para definir a periodontite, bem como do desenho do estudo, da técnica empregada para avaliar a presença de periodontopatógenos, e as espécies específicas de bactérias avaliadas.

Uma comparação das características clínicas e demográficas das participantes do presente estudo encontrou homogeneidade em relação a maioria dos aspectos que representam fatores de risco para complicações gestacionais. No entanto, alguns fatores de alto risco foram observados com maior frequência em mães de bebês com BPN. A análise multivariada foi empregada para ajustar idade, IMC pré-gestacional e renda familiar, a fim de neutralizar o efeito dessas covariáveis de confusão no grupo estudado. No entanto, nenhuma significância estatística foi observada. Esse achado está de acordo com análises anteriores, ^( 5 )^ que não encontraram diferenças significativas, mesmo após ajuste de fatores de confusão, nos níveis de periodontopatógenos entre mulheres sem desfechos gestacionais adversos, bem como naquelas que tiveram parto prematuro e/ou naquelas que deram à luz recém-nascidos com BPN.

Apesar da falta de significância estatística neste estudo para a associação entre escolaridade materna e BPN, não se pode desconsiderar que o nível de escolaridade e a percepção corporal influenciam no grau de conhecimento sobre os cuidados em saúde. ^( 24 )^

Este estudo encontrou alta prevalência de periodontopatógenos em ambos os grupos, o que Dumitrescu et al., ^( 25 )^ chamaram de “estado portador”, em que baixas quantidades de periodontopatógenos podem estar presentes em indivíduos sem diagnóstico clínico de periodontite. Isso complica ainda mais a avaliação de seu papel nessa doença.

Como várias espécies de bactérias periodontais são comumente encontradas juntas em bolsas periodontais, o isolamento de um tipo específico responsável pela patogênese dessa doença pode ser tarefa complexa. ^( 25 )^ Devido à interação e à cooperação evidenciadas entre os diversos periodontopatógenos, ^( 26 , 27 )^ também foi avaliada a ocorrência de coinfecção entre as mulheres estudadas.

Nossos resultados demonstram que a presença de três ou mais patógenos em mulheres grávidas não está associada a bebês com BPN. No entanto, diferentemente das análises de OR, considerando cada patógeno separadamente, a medida da associação estatística em relação à coinfecção se aproxima do nível de significância.

O uso recente de técnicas de sequenciamento metagenômico permite a triagem da composição genética e do potencial funcional de uma comunidade microbiana, fornecendo visão abrangente dessas comunidades em associação com a saúde periodontal e a periodontite crônica. ^( 28 )^ Além disso, pode haver várias cepas de cada espécie periodontopatogênica com fatores de virulência de potencial variável. A capacidade que cada uma dessas espécies tem de translocar, invadir e colonizar tecidos fetais, bem como de evitar respostas imunológicas do hospedeiro, determina seu potencial específico de contribuir para desfechos adversos da gravidez. ^( 29 )^ Portanto, são necessários mais estudos para investigar a heterogeneidade das comunidades microbianas periodontais em indivíduos.

É sabido que as bactérias, por si só, não são capazes de provocar destruição tecidual grave em todos os indivíduos, o que sugere que os indivíduos têm capacidade variável de se adaptarem a uma determinada carga bacteriana contida em seu biofilme. ^( 30 )^

Os achados do presente estudo não indicam que a presença de um periodontopatógeno específico em si possa ser um fator biológico plausível na associação entre periodontite materna e a ocorrência de bebês com BPN. Por outro lado, o presente estudo não apenas reafirma a associação entre periodontite materna e bebê com BPN, mas também amplia o conhecimento atual, ao fornecer amostra substancialmente maior, permitir a exploração de possíveis fatores de confusão, aumentar o número de locais de coleta de biofilme e considerar um modelo de coinfecção na análise. No entanto, mais estudos são necessários para incluir um número maior de subgrupos de coinfecção, empregando amostras maiores.

## CONCLUSÃO

Nesta população, não houve associação entre a presença de agentes patogênicos periodontais e a ocorrência de recém-nascidos com baixo peso ao nascer.
